# Protective biomechanical and histological changes in the false lumen wall in chronic type B aortic dissection

**DOI:** 10.1016/j.xjon.2024.11.012

**Published:** 2024-11-30

**Authors:** Hai Dong, Minliang Liu, Hannah L. Cebull, Arshiya Chhabra, Yumeng Zhou, Marina Piccinelli, John N. Oshinski, John A. Elefteriades, Rudolph L. Gleason, Bradley G. Leshnower

**Affiliations:** a Carlyle Fraser Cardiothoracic Research Laboratory, Division of Cardiothoracic Surgery, Emory University School of Medicine, Atlanta, Ga; b The George W. Woodruff School of Mechanical Engineering and The Wallace H. Coulter Department of Biomedical Engineering, Georgia Institute of Technology, Atlanta, Ga; c Department of Mechanical Engineering, Texas Tech University, Lubbock, Tex; d Department of Radiology and Imaging Science, Emory University School of Medicine, Atlanta, Ga; e Division of Cardiac Surgery, Aortic Institute at Yale-New Haven Hospital, Yale University School of Medicine, New Haven, Conn

**Keywords:** aortic dissection, false lumen wall, biomechanical stiffness, histological analysis

## Abstract

**Objective:**

The outer false lumen wall (FLW) changes from thin/compliant to thick/rigid as aortic dissection transitions from the acute to chronic phase. This study investigates biomechanical stiffness and histological changes of the FLW as the dissected aorta ages.

**Methods:**

The free outer FLW from human tissue was analyzed from chronic type B dissection (chronic-FLW) n = 10, acute type A dissection (acute-FLW) n = 10, and transplant donor descending aorta that was manually peeled into 2 layers (control-FLW) n = 17. Biaxial tension testing in the longitudinal and circumferential directions was performed and stress-strain curves were obtained. A lower and higher tangent modulus was determined to assess stiffness. Quantification of collagen and elastin was performed by calculating the fibers’ volume fraction from Z-stack scans.

**Results:**

The higher tangent modulus of chronic-FLW is larger (*P* < .01) than the acute-FLW and control-FLW in longitudinal (5.09 ± 0.9 MPa vs 1.72 ± 0.56 MPa and 1.17 ± 0.22 MPa) and circumferential (4.16 ± 0.67 MPa vs 1.04 ± 0.24 MPa and 1.07 ± 0.16 MPa) directions. The lower tangent modulus of chronic-FLW is larger (*P* < .05) than acute-FLW and control-FLW in both directions (longitudinal: 0.72 ± 0.24 MPa vs 0.13 ± 0.02 MPa and 0.27 ± 0.03 MPa circumferential:0.44 ± 0.13 MPa vs 0.12 ± 0.01 MPa and 0.21 ± 0.02 MPa). The volume fraction of collagen was increased (*P* < .01) and the volume fraction of elastin was decreased (*P* < .001) when comparing chronic-FLW, acute-FLW, and control-FLW (collagen-volume fraction: 0.24 ± 0.03 vs 0.12 ± 0.03 and 0.08 ± 0.02; elastin-volume fraction: 0.09 ± 0.03 vs 0.28 ± 0.03 and 0.39 ± 0.04).

**Conclusions:**

As the acutely dissected aorta transitions to the chronic phase, the FL remodels by increasing collagen, decreasing elastin, and increasing aortic stiffness and thickness. This change in the chronic-FLW may be a protective adaptation to prevent FL enlargement and rupture in type B aortic dissection.

## Graphical Abstract

**Figure:**
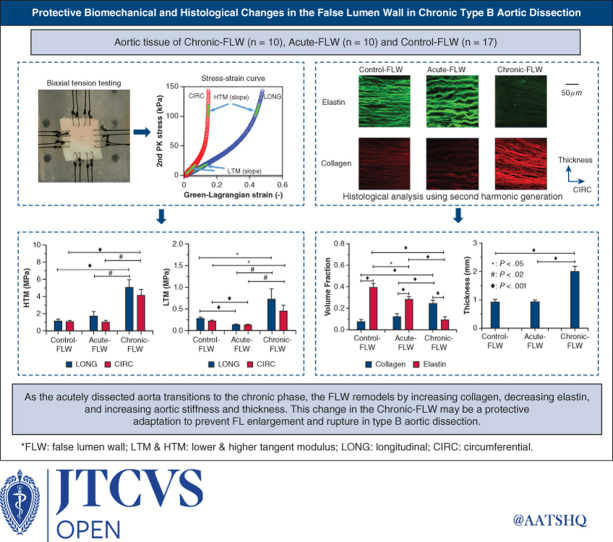

**Figure:**
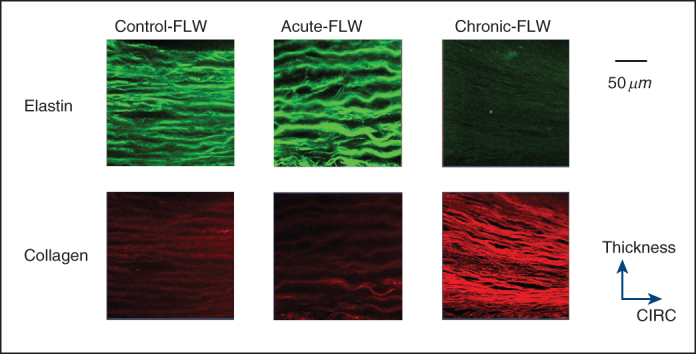
Less elastin and more collagen in chronic-FLW compared with control-FLW and acute-FLW.

**Central Message**

As the acutely dissected aorta transitions to the chronic phase, the FL remodels by increasing collagen and decreasing elastin. This metamorphosis results in increased aortic stiffness and thickness.

**Perspective**

This study is the first to characterize the temporal changes that occur in the FL wall as the aorta transitions from the acute to the chronic phase. Based on our findings, the FL wall remodels over time and becomes thicker and stiffer. This metamorphosis occurs due to a change in the profile of the FL wall microstructure, including the accumulation of collagen and degradation of elastin.

Type B aortic dissection (TBAD) is a life-threatening event that is initiated by a tear in the intimal layer of the aortic wall distal to the left subclavian artery. The tear is the result of a mechanical failure in which aortic wall stress exceeds aortic wall strength. Blood flow into the tear propagates the dissection and delaminates the media, dividing the single lumen aorta into a blood vessel with 2 lumens. The true lumen has a normal trilayer outer wall composed of intima, media, and adventitia and shares a common partial thickness (intimal-medial) wall (dissection flap) with the false lumen (FL) that has an outer aortic wall composed of partial thickness media and adventitia alone. In the acute phase of TBAD, the FL wall (FLW) is thin and fragile, which makes the aorta susceptible to rapid enlargement and rupture within the first postdissection month.^1^ Transition into the chronic phase is accompanied by lower growth and rupture rates, yet FL aneurysm formation occurs in 73% of patients with TBAD at 5 years despite medical therapy.^1^^,^^2^

The reduction in growth and rupture rates as the dissected aorta transitions from the acute to chronic phase can be attributed to either decreased FL stress and/or increased FLW strength. The stress or load applied to the FLW is primarily influenced by blood pressure and can be reduced with antihypertensiontherapy. However, a detailed understanding of the FLW mechanical properties (stiffness/strength) is lacking. Investigations are needed into the biomechanical and histological changes that occur as the FLW remodels. Contrary to linear elastic materials like steel, in which the stiffness can be characterized by a single parameter (eg, Young's modulus), the material mechanical properties of the aorta are complex. The stiffness of aortic tissue cannot be adequately described by a single parameter due to its inherently nonlinear and anisotropic mechanical behaviors.^3^, ^4^, ^5^, ^6^, ^7^, ^8^ Specifically, the stress-strain curve of aortic tissue typically exhibits a *J*-shape, with increased stiffness at higher levels of force applied to the wall (nonlinear). Also, the deformation responses of the aortic wall differ depending upon the direction of force (anisotropic).

It is well recognized that the mechanical properties of the dissection flap and FLW change from thin and compliant to thick and rigid, as the dissected aorta transitions from the acute to chronic phase in TBAD.^1^ These differences are evident in the surgical treatment of aortic dissection. In the acute phase, the FLW is fragile, does not hold sutures, and must be reapproximated to the dissected intimal-medial (dissection flap) layer to obtain hemostasis with a surgical graft anastomosis. In the repair of chronic dissection, the FLW is thick, holds sutures well, and has sufficient integrity to allow direct anastomosis with a surgical graft without reapproximation or incorporation of the intimal-medial layer. The purpose of this study was to characterize the histologic and biomechanical changes that occur in the dissected aortic wall, as the aorta progresses from the acute to chronic phase—to improve our understanding of the complex behavior of the FLW.

## Materials and Methods

This retrospective study was approved by the Emory University Institutional Review Board (IRB00109646 and STUDY00002737; July 23, 2021). Patients’ informed written consents were obtained for the publication of the study data.

### Tissue Collections

Ascending and descending/thoracoabdominal aortic replacements were performed using standard surgical techniques. Surgical specimens were removed from the field, marked for orientation, placed into cryopreservation medium (10% dimethylsulfoxide + 90% Roswell Park Memorial Institute medium), transported to the tissue processing lab, and stored in a −80 °C freezer. Currently, the acute type A dissection is a surgical emergency and requires heart surgery and ascending aortic replacement immediately after diagnosis. Thus, patients with acute type A dissection who do not undergo emergency surgery and survive to the chronic phase are rare. In contrast, the current standard treatment for TBAD is either medical therapy or thoracic endovascular aortic repair, and therefore there is limited access to acute type B tissues. Surgical specimens of the FLW were analyzed from 10 patients with chronic TBAD (chronic-FLW), and from 10 patients with acute type A aortic dissections (acute-FLW), who underwent open aortic replacement at Emory University Hospital. The chronic-FLW samples were excised at a mean duration of 38.9 ± 8.9 months after the dissection occurred, while the acute-FLW samples were excised within 12 hours of the patients diagnosed with type A dissection. The third group of tissues tested were descending thoracic aortic specimens obtained from 17 human transplant donors without aortic dissection or aneurysmal disease. To serve as a control group to compare against the acute-FLW and chronic-FLW specimens, the donor aortic tissue was manually peeled into two layers by delaminating the media. The outer layer (control-FLW: containing partial media and adventitia) was taken for comparisons with the chronic-FLW and acute-FLW.

### Biaxial Tension Testing

**Figure 1:**
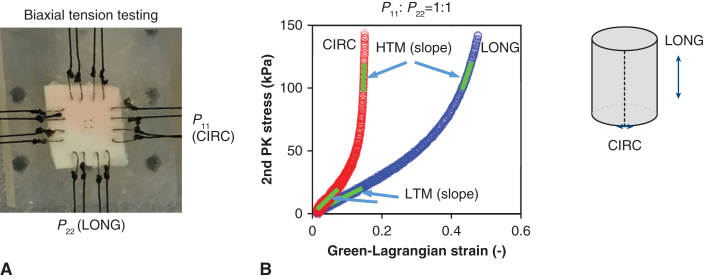
A, A representative sample under biaxial tension testing in circumferential (*CIRC*) and longitudinal (*LONG*) directions. B, Stress-strain curve under equal-loading (*P*_11_:*P*_22_=1 ∶ 1) was obtained. *HTM*, Higher tangent modulus; *LTM*, lower tangent modulus.

The tissue was thawed at around 37 °C before testing. Planar biaxial tension testing in the circumferential and longitudinal directions was performed on each of the FLW samples from the 3 groups (control-FLW, acute-FLW, and chronic-FLW), based on the well-established methods.^9^, ^10^, ^11^ Briefly, a square section of the FLW (Figure 1, *A*) was delimited by 16 suture hooks, 4 per side. Four graphite markers were affixed with cyanoacrylate adhesive to delineate a 2 × 2 mm square in the center of the testing area for strain tracking. The sample was mounted onto a testing machine in a trampoline-like fashion, and submerged in a 0.9% saline solution kept at 37 °C for the duration of the test. The circumferential direction was aligned with the *X*_1_ testing axis, and the longitudinal direction with the *X*_2_ axis. A stress-controlled testing protocol was applied, with the ratio of the normal stress components *P*_11_:*P*_22_ predefined and with the shear terms *P*_12_=*P*_21_=0. A stress-strain curve (Figure 1, *B*) under equibiaxial loading (*P*_11_:*P*_22_=1 ∶ 1) was obtained for each of the samples.

### Tissue Stiffness Analysis

The stress-strain curve of the FLW tissue is nonlinear and exhibits a *J*-shape with greater stiffness at larger deformation. The mechanical responses are different in the circumferential and longitudinal directions (Figure 1, *B*). Therefore, to accurately characterize the tissue stiffness, a lower tangent modulus (LTM) and higher tangent modulus (HTM) were determined for each sample in the circumferential and longitudinal directions, based on the low and high linear regions of the equibiaxial testing stress-strain curves (Figure 1, *B*). Specifically, the LTM and HTM were obtained by fitting the data points within each region (green lines in Figure 1, *B*) in the least-square sense by means of a custom MATLAB 2018b code (MathWorks Inc).

### Histology

**Figure 2:**
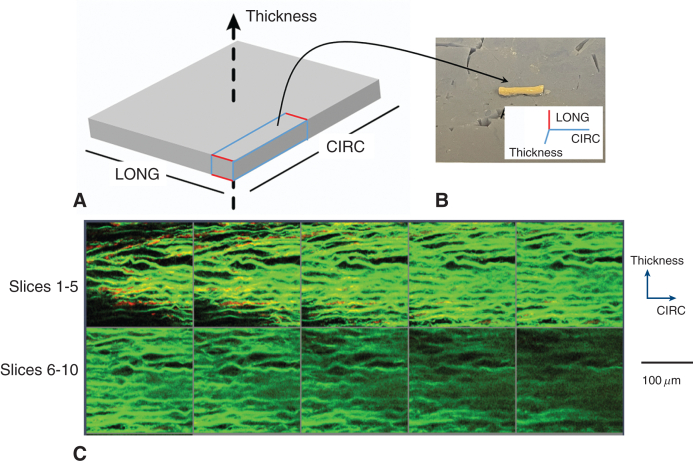
A, A schematic for an false lumen wall tissue sample. B, A tissue strip cut from the whole tissue for microscopic observation. C, Image slices 1 through 10 of a representative Z-sack scan with a step of 5 μm and a depth of ∼50 μm. *LONG*, Longitudinal; *CIRC*, circumferential.

A histologic analysis of the tissue microstructure was performed on the collagen and elastin fibers using second harmonic generation (SHG) microscopy. A total of 15 FLW samples were imaged (n = 5 for each group). A strip sample (Figure 2, *B*) was cut from the FLW tissues (Figure 2, *A*) and was imaged on a Zeiss 710 NLO inverted confocal microscope (Carl Zeiss Microscopy, LLC). A mode-locked Ti: Sapphire Chameleon Ultra laser (Coherent Inc) was applied using non-descanned detection. The laser was set to 800 nm and emission was filtered from 380 to 430 nm for collagen fibers,^12^ and filtered from 500 to 550 nm for elastin fibers.^13^^,^^14^ SHG images were collected using a Plan-Apochromat 40× oil immersion objective.

The Z-stack scan (Figure 2, *C*) was obtained along the longitudinal (LONG) direction of the tissue (perpendicular to the circumferential [CIRC-Thickness plane]) (Figure 2, *B*) with a step of 5 μm and a depth of ∼50 μm. For each sample, 4 to 9 Z-stack scans (based on thickness) were obtained at different locations along the thickness direction within the CIRC-Thickness plane. Quantification of the composition of collagen and elastin fibers was performed by calculating the fibers’ volume fraction (φ) from the Z-stack scans defined as

$$\varphi =\frac{V_{\text{fiber}}}{V_{\text{total}}}=\frac{\sum_{i=1}^{N}A_{\text{fiber}}∙L_{\text{step}}}{A_{\text{total}}∙L_{\text{total}}}\text{,}$$

where V_fiber_ is the collagen/elastin fiber volume, V_total_ is the total volume, A_fiber_ is the area of the collagen/elastin fiber in a specific slice image, L_step_ = 5 μm is the step distance between the neighboring slice images, A_total_ is the total area of the slice image, and L_total_ is the distance between the first and last slice images.

### Statistical Analysis

Two-sample Kolmogorov-Smirnov tests were used to compare the parameters from different groups. All analyses were performed with the *kstest2* function in MATLAB 2024ab.

## Results

### Tissue Stiffness

**Figure 3:**
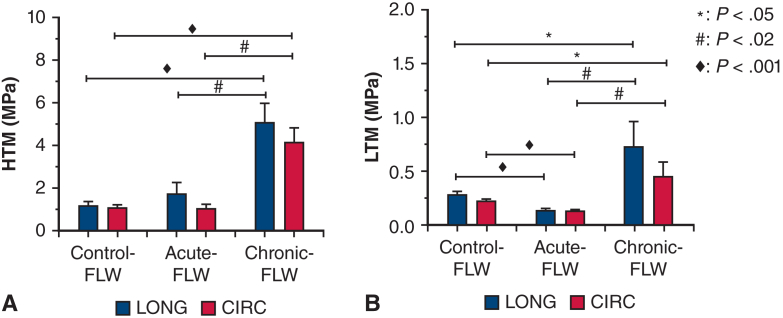
Mean ± SE of the higher tangent modulus (*HTM*) (A) and lower tangent modulus (*LTM*) (B) of the control-false lumen wall (*FLW*), acute-FLW, and chronic-FLW, in circumferential (*CIRC*) and longitudinal (*LONG*) directions.

The HTM of the chronic-FLW is significantly larger than those of the acute-FLW and the control-FLW in both longitudinal (mean ± SE, 5.09 ± 0.91 MPa vs 1.72 ± 0.56 MPa [*P* = .007] and 1.17 ± 0.22 MPa [*P* < .001], respectively) and circumferential (4.16 ± 0.67 MPa v 1.04 ± 0.24 MPa [*P* = 0 .001] and 1.07 ± 0.16 MPa [*P* < .001], respectively) directions (Figure 3, *A*). No statistical difference was found for the HTM between control-FLW and acute-FLW (*P* > .05). The LTM of the chronic-FLW is also larger in comparison with those of the acute-FLW and the control-FLW in both directions (Figure 3, *B*) (LONG: 0.72 ± 0.24 MPa vs 0.13 ± 0.02 MPa [*P* = .005] and 0.27 ± 0.03 MPa [*P* = .036]; CIRC: 0.44 ± 0.13 MPa vs 0.12 ± 0.01 MPa [*P* = .017] and 0.21 ± 0.02 MPa [*P* = .028]). For the LTM, the control-FLW is also larger than the acute-FLW in both directions (Figure 3, *B*) (both *P* values < .001).

### Histology: Microstructure and Volume Fraction of Collagen and Elastin Fibers

**Figure 4:**
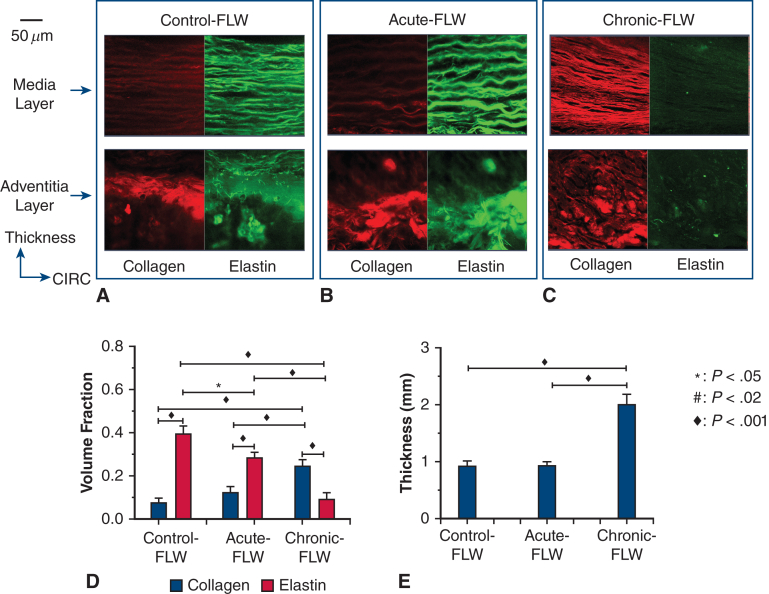
A through C, Representative examples of the microstructure of the collagen and elastin fibers in the control-false lumen wall (*FLW*) (A), acute-FLW (B), and chronic-FLW (C) tissues. D, Mean ± SE of the volume fraction of the collagen and elastin fibers in the tissue of the 3 groups. E, Mean ± SE of the thickness of the tissue of the 3 groups. *CIRC*, Circumferential.

For a qualitative characterization (Figure 4, *A-C*), the SHG microscopy demonstrated similar appearances of the control-FLW and acute-FLW. Both of these groups had highly concentrated, well-organized layers of elastin in a parallel distribution in the medial layer of the FLW, with scant, but well-organized layers of collagen. In the adventitial layer, there appeared to be equal concentrations of elastin and collagen, with both proteins in a poorly oriented configuration (Figure 4, *A* and *B*). The chronic-FLW had a dramatically different appearance. In the medial layer, there was a high concentration of collagen that was well organized in a parallel distribution with a low concentration of elastin. In the adventitial layer, there was a high concentration of poorly organized collagen and minimal elastin present (Figure 4, *C*).

Quantification of the collagen/elastin profile in the tissue demonstrated a significant increase in the volume fraction (VF) of collagen fibers and a significant decrease in the VF of elastin fibers when comparing the chronic-FLW versus the acute-FLW and control-FLW (Figure 4, *D*) (collagen-VF: 0.24 ± 0.03 MPa vs 0.12 ± 0.03 MPa [*P* < .001] and 0.08 ± 0.02 MPa [*P* < .001], respectively; elastin-VF: 0.09 ± 0.03 MPa vs 0.28 ± 0.03 MPa [*P* < .001] and 0.39 ± 0.04 MPa [*P* < .001], respectively). The VF of collagen fibers is larger than that of elastic fibers within the chronic-FLW (*P* < .001), but smaller than that of elastin fibers within the acute-FLW (*P* < .001) and control-FLW (*P* < .001) (Figure 4, *D*). The thickness of the chronic-FLW is significantly larger than those of the control-FLW and acute-FLW (Figure 4, *E*) (1.99 ± 0.19 mm vs 0.92 ± 0.08 mm [*P* < .001] and 0.91 ± 0.09 MPa [*P* < .001], respectively), which may be due to the accumulation of the collagen fibers as the aorta remodels in the chronic phase.

**Figure 5:**
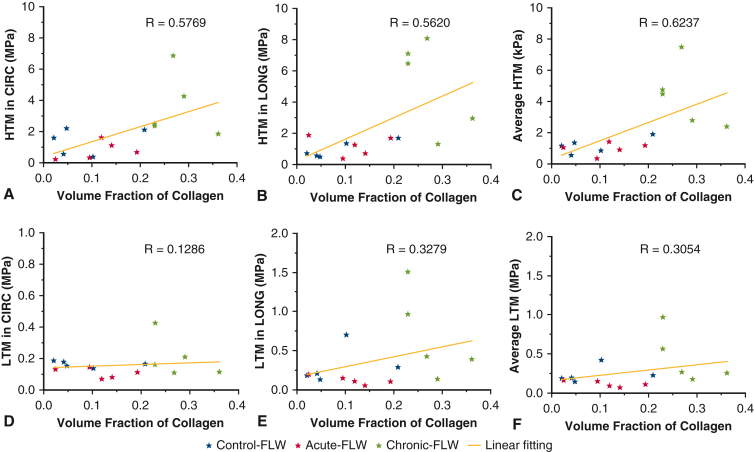
Linear regression between the volume fraction of collagen fibers and the higher tangent modulus (*HTM*) (A-C) and lower tangent modulus (*LTM*) (D-F) tangent modulus, in circumferential (*CIRC*) and longitudinal (*LONG*) directions, and 2-direction average. *FLW*, False lumen wall.

**Figure 6:**
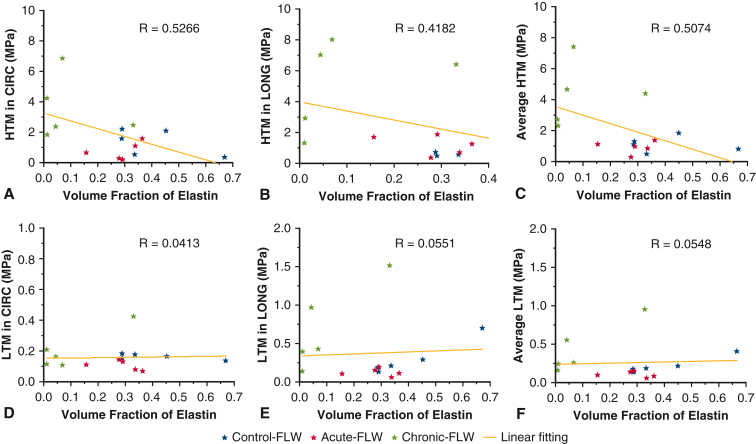
Linear regression between the volume fraction of elastin fibers and the higher tangent modulus (*HTM*) (A-C) and lower tangent modulus (D-F), in circumferential (*CIRC*) and longitudinal (*LONG*) directions, and 2-direction average. *FLW*, False lumen wall; *LTM*, lower tangent modulus.

Linear regression showed that the HTM in both CIRC and LONG directions has a positive correlation with the VF of collagen fibers (Figure 5, *A-C*), and a negative correlation with the VF of elastin fibers (Figure 6, *A-C*). The LTM has less correlation with the VF of collagen/elastin fibers (Figure 5, *D-F*, and Figure 6, *D-F*). The larger VF of collagen and lower VF of elastin in chronic-FLW are likely responsible for the increased stiffness of the chronic-FLW compared with the acute-FLW and control-FLW.

### Age Analysis

It is well known that age influences the stiffness of aortic tissues, generally causing them to stiffen as they age.^15^^,^^16^ We analyzed the age information and found no statistical difference in the age between the 3 groups (control-FLW: 48.2 ± 4.7 years, acute-FLW: 49.9 ± 3.44 years, and chronic-FLW: 45.2 ± 4.7 years; a ll *P* values > .05 between each pair of groups). Thus, the increased stiffness observed in the chronic-FLW compared with the acute-FLW and control-FLW may not be attributed to aging effects.

## Discussion

Current knowledge is lacking regarding the temporal changes that occur in the FLW as the acutely dissected aorta transitions to the chronic phase. In the majority (67%) of patients with aortic dissection, the ascending aorta is involved (type A) and emergency surgical replacement is warranted. A small percentage of these patients survive to the chronic phase with medical therapy alone. In sharp contrast, the majority of patients with acute TBAD are uncomplicated (no malperfusion or rupture) and have excellent survival with medical therapy as they transition to the chronic phase (>3 months). In these patients, the rate of FL enlargement and aortic rupture peaks within the first month postdissection, but is significantly reduced by the 3-month mark, as the dissection enters the chronic phase.^1^ In this study, we provide biomechanical and histologic data to account for the reduction in the rate of FL expansion/rupture. Our investigations demonstrate that the FLW remodels and becomes stiffer primarily due to a reversal in the elastin/collagen profile of the FLW microstructure.

Ex vivo tensile testing of aortic tissue remains the gold standard to evaluate the mechanical properties of the aortic wall. To assess the biomechanical changes of the FLW that occur as the dissected aorta ages, we performed biaxial tensile testing on aortic specimens with acute and chronic dissection. Testing was performed on descending thoracic aorta from transplant donors that were manually peeled to create a dissection (control-FLW), acutely dissected ascending aorta from acute type A repairs (acute-FLW), and chronically dissected aortic specimens (chronic-FLW) from descending/thoracoabdominal aneurysm repairs secondary to chronic distal dissection. The control-FLW group was tested to account for any differences in the mechanical properties between the ascending and descending aorta. The main findings of the biaxial tensile testing were that the higher tangent modulus and lower tangent modulus of the chronic-FLW were significantly larger than those of the acute-FLW and control (normal aorta) tissues in both the LONG and CIRC directions (Figure 3). These findings signify an increased resistance to deformation; that is, increased tissue stiffness, in the chronic-FLW at both high and low levels of stress in both the LONG and CIRC directions. It should also be noted that there was no difference in the HTM between the control-FLW and acute-FLW in the circumferential and longitudinal directions, but the LTM was greater in the control-FLW than the acute-FLW in both directions. Interpretation of these findings is challenging and could be attributed to a true difference in the media layer of the aortic wall in the ascending and descending aorta due to the location effect,^17^^,^^18^ because the media is the primary load bearing layer at lower levels of stress.^19^ Alternatively, it may be that the manual shearing within the media for the control-FLW does not adequately replicate the fine details of a native dissection (eg, vis-à-vis the depth of the shearing plane in the natural vs the experimental shearing process).

Once these changes in FLW mechanics were identified, we performed a histologic analysis of the microstructure of the FLW to elucidate the mechanism behind the increased tissue stiffness. SHG imaging demonstrated significant differences between the chronic-FLW specimens compared with the acute-FLW and control-FLW specimens. The media layer of the acute-FLW and control-FLW specimens had highly concentrated, well-organized layers of elastin and scant, disorganized collagen layers. In sharp contrast, the media layer of the chronic-FLW had a high concentration of well-organized collagen and a low concentration of elastin. The high collagen to elastin ratio in the microstructure was also present in the adventitial layer of the chronic-FLW, as opposed to relatively equal concentrations of the 2 proteins in the acute-FLW and control-FLW specimens (Figure 4, *A-C*). Using a VF analysis, we quantified the amount of collagen and elastin in the aortic specimens (Figure 4, *D*). Acute-FLW and control-FLW specimens had significantly higher concentrations of elastin relative to collagen. This relationship was reversed in the chronic-FLW, with a significantly higher concentration of collagen compared with elastin. The increased thickness of the chronic-FLW (Figure 4, *E*) also reflects the increased collagen to elastin ratio because the diameter of a collagen fiber is 10× greater than the diameter of elastin fiber.^20^

Previous investigations into the mechanical properties of the thoracic aorta have largely focused on aneurysms, with only a limited amount of work being performed in aortic dissection tissue. The few existing reports did demonstrate increased tissue stiffness in the dissected tissue. Babu and colleagues^21^ demonstrated a higher stiffness in human acute type A aortic dissection tissue compared with control aorta with no correlation to diameter or age; however a layer-specific analysis was not performed.^21^ Deplano and colleagues^22^ compared the mechanical properties of normal human aorta to the different layers of the acutely dissected ascending aorta from a single patient. These investigators reported increased stiffness in the dissected aorta; however, they were unable to perform tensile testing on the adventitial (outer FLW) layer due to technical difficulties.^22^ Two additional case reports on chronic type A aortic dissection tissue using biaxial tensile testing reported increased stiffness in the false lumen wall compared with surrounding aneurysmal tissue at higher levels of wall stress.^23^^,^^24^ There have been no reports investigating the biomechanical and histologic changes that occur as the aorta remodels and transitions from the acute to chronic phase of dissection.

The main limitation of this study is the lack of acutely dissected descending aortic specimens. Given that endovascular therapy is the current first line therapy for acute complicated acute TBAD, there is an insufficient number of acutely dissected descending aortic specimens in our aortic tissue bank. Therefore, we used acutely dissected ascending aortic specimens from patients with acute type A dissection as a surrogate. Considering that the ascending and descending aortas have distinct embryologic origins—the ascending aorta develops from the primordial aortic sac and truncus arteriosus, whereas the descending aorta arises from the fusion of the right and left dorsal aortas below the T4 vertebral level^25^— these differences may influence their collagen/elastin content and material properties. This introduces the possibility that the mechanical properties of the ascending and descending aorta are significantly different and therefore, influence the results of the study. Moreover, it remains unknown whether patients, who survive acute TBAD with optimal medical therapy, have an inherently stiffer aorta that allows their survival into the chronic phase. Future studies may directly investigate the in vivo tissue stiffness using noninvasive measurement techniques. An additional limitation is related to the aortic peeling process in the healthy control aortic tissue. The aortic wall is manually peeled into 2 layers by dividing the media. This iatrogenic dissection could produce a simulated FLW (control-FLW) that may be different in thickness compared with a natural dissection. However, in this work, the control-FLW may still be comparable to the FLW of an acute aortic dissection because the thickness of the control-FLW has no statistical difference from the acute-FLW (Figure 4, *C*). Moreover, here we investigated elastin and collagen because it is well known that these 2 fibers are among the most important components that influence the biomechanical properties of aortic tissues.^26^ Future investigations may include other components such as smooth muscle cells. Finally, other patient characteristics such as body mass index, comorbidities, and previous surgery may also influence the results, which may be investigated in future studies.

To our knowledge, this is the first study to characterize the temporal changes that occur in the FLW as the aorta transitions from the acute to the chronic phase. Based on our findings, the FLW remodels over time and becomes thicker and stiffer. This metamorphosis occurs due to a change in the profile of the FLW microstructure, namely collagen and elastin. These 2 proteins are the principal determinants of the mechanical properties of the aortic wall, with collagen being primarily responsible for both stiffness and tensile strength, particularly at high loads of stress.^27^ We demonstrated that as the acutely dissected aorta ages, the collagen content of the media and adventitial layers of the FLW increases and becomes more organized, whereas the elastin content is substantially reduced. Collagen organization and orientation have also been shown to influence aortic wall strength.^28^^,^^29^ These findings are similar to the effect of normal aging on the aortic wall, in which there is an accumulation of collagen and a degradation of elastin that results in the stiffening of the aortic tissue.^15^^,^^16^ The remodeling process in dissection may be interpreted as an accelerated aging effect that is protective against rapid expansion and/or rupture of the FL.

In essence, during the weeks and months after the acute dissection, the aorta protects itself against rupture via this strengthening of outer (adventitial) layer of the aortic wall. These detailed biomechanical and histologic studies have confirmed what Griepp, Cooley, and others gleaned in the pioneering days of aortic surgery: Operating on an acutely dissected descending aorta is fraught with danger; however, open surgery on the descending aorta during the chronic phase is safe. The biological/mechanical compensations enacted by the aorta that are disclosed by our investigations explain scientifically what the surgical pioneers knew from intuitive acumen.

## Conclusions

This study demonstrated that the biomechanical stiffness of the FLW increased as the aorta remodels and transitions from acute to chronic phase. This remodeling process was due to a change in the microstructure of the FLW with the accumulation of collagen fibers and degradation of elastin fibers, which also results in the increase of the thickness compared with acute and control samples. The change in the composition, thickness, and stiffness of the FLW tissue may be a protective adaptation to prevent rapid FL expansion and/or rupture in chronic TBAD.

## Conflict of Interest Statement

Dr Leshnower is a is a consultant for Endospan Inc and speaker for Medtronic. Dr Elefteriades is principal of CoolSpine. All other authors reported no conflicts of interest.

The *Journal* policy requires editors and reviewers to disclose conflicts of interest and to decline handling or reviewing manuscripts for which there may be a conflict of interest. The editors and reviewers of this article have no conflicts of interest.
